# miR-223: An Effective Regulator of Immune Cell Differentiation and Inflammation

**DOI:** 10.7150/ijbs.59876

**Published:** 2021-06-04

**Authors:** Peng Jiao, Xing-Ping Wang, Zhuo-Ma Luoreng, Jian Yang, Li Jia, Yun Ma, Da-Wei Wei

**Affiliations:** 1 School of Agriculture, Ningxia University, Yinchuan 750021, China.; 2 Key Laboratory of Ruminant Molecular Cell Breeding, Ningxia Hui Autonomous Region, Yinchuan 750021, China.

**Keywords:** miR-223, Cellular inflammation, Inflammatory diseases, Immunity, Molecular regulatory network

## Abstract

MicroRNAs (miRNAs) play a critical role in regulating various biological processes, such as cell differentiation and immune modulation by binding to their target genes. miR-223 is a miRNA with important functions and has been widely investigated in recent years. Under certain physiological conditions, miR-223 is regulated by different transcription factors, including sirtuin1 (Sirt1), PU.1 and Mef2c, and its biological functions are mediated through changes in its cellular or tissue expression. This review paper summarizes miR-223 biosynthesis and its regulatory role in the differentiation of granulocytes, dendritic cells (DCs) and lymphocytes, macrophage polarization, and endothelial and epithelial inflammation. In addition, it describes the molecular mechanisms of miR-223 in regulating lung inflammation, rheumatoid arthritis, enteritis, neuroinflammation and mastitis to provide insights into the existing molecular regulatory networks and therapies for inflammatory diseases in humans and animals.

## Introduction

Inflammatory diseases are commonly caused by pathogen infection or external factors (e.g., trauma) 1. Chronic inflammation could result in metabolic disorders, organ damages, and severe inflammatory diseases that could even cause human or animal death. Therefore, exploring different molecular mechanisms on the development and progression of inflammatory diseases could improve the breeding of new animal varieties with strong disease resistance. Besides, it could help develop early diagnostic tools and biological or chemical therapies for human inflammatory diseases. Notably, many factors could trigger inflammatory diseases. Recent studies have shown that the development and progression of inflammatory diseases are very complex and are regulated by a molecular network involving multiple genes or proteins 2, 3. miRNAs are a newly identified class of non-coding RNAs that could influence immune responses, cancer development, and cell proliferation, differentiation, and apoptosis by regulating the expression of target mRNAs 2. Monitoring their level is critical for early disease diagnosis and prognostic observation 3. Many researchers have screened and identified inflammation-related miRNAs, and conducted their functional analyses in human and animal cells and tissue inflammation. Of these miRNAs, miR-223 has been identified to exhibit multiple regulatory functions during inflammation 4. This review paper has discussed the modulatory effects of miR-233 in the differentiation of multiple cell types and inflammation. Also, it provides a systematic overview of the molecular mechanisms of miR-223 in regulating lung inflammation, rheumatoid arthritis, enteritis, neuroinflammation and mastitis to offer new insights into treatment development for inflammatory diseases in humans and animals.

## Biosynthesis of miR-223

**Figure 1:**
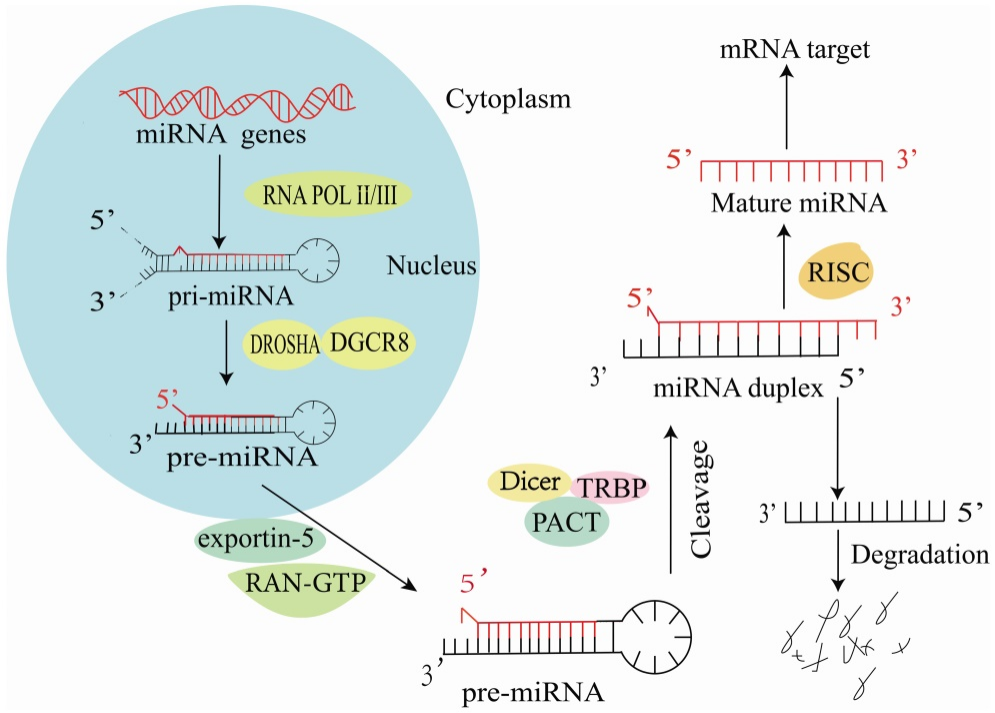
Synthesis of miR-223 in cells.

Mature miRNAs are short non-coding RNA molecules comprising 19-25 nucleotides 5. In the nucleus, RNA polymerase II synthesizes most miRNAs into primary miRNAs (pri-miRNAs) 6 and most miRNAs are subsequently cleaved by Drosha RNase III into 70-80 nt long hairpin precursor miRNAs (pre-miRNAs) 7. Next, exportin-5 transports pre-miRNAs into the cytosol, where they are cleaved by the endoribonuclease Dicer 8 to form double-stranded RNA molecules. These double-stranded RNA molecules are modified by the RNA-induced silencing complex (RISC), and one strand is retained to target and regulate mRNA expression (**Figure 1**) 9.

miR-223 is an important member of the miRNA family, first identified by quantitative polymerase chain reaction (qPCR) in 2003 10. Its genes are located in the X chromosome of humans, mice and cows. Under certain physiological conditions, miR-223 expression is promoted by different transcription factors, C/EBPα, PU.1 and C/EBPβ 11, 12 and inhibited by nuclear factor I A (NFIA), Mef2c and KLF6 13-17, and the importin-α4 and importin-α5 transporters 18.

## miR-223 expression during cellular and tissue inflammation

Several studies have been conducted to understand the miR-223 functions that determine its cellular and tissue expression 13, 15, 19-22. Based on these studies, miR-223 expression is altered during the inflammatory response of various cell types, including granulocytes, macrophages, dendritic cells (DCs), T cells, endothelial cells and epithelial cells. This change in miR-223 expression regulates the various functions of these cells and attenuates or exacerbates the associated tissue inflammation (**Table 1**).

## Molecular mechanism of action of miR-223 at the cellular level

miR-233 regulates the differentiation and proliferation of granulocytes, macrophages and DCs by binding to specific targets. In addition, miR-223 regulates pro-inflammatory or anti-inflammatory macrophage polarization. miR-223 can also bind specific target genes to inhibit pro-inflammatory cytokines or inflammatory signals in these cells (**Table 2**).

### Role of miR-223 in granulocyte differentiation

miR-223 plays a critical role in the differentiation and activation of granulocytes. According to Johnnidis et al. 13 miR-223 could target the myocyte enhancer factor 2C (Mef2c) to regulate neutrophil progenitor proliferation and granulocyte differentiation and activation in mice. miR-223-deficient mice had hyperactive granulocytes that were highly sensitive to activating stimuli 13. Additionally, the lipopolysaccharide (LPS) challenge resulted in rapid accumulation of endotoxin, and the inflammatory lung pathology induction was characterized by excessive lung tissue injury 13. During Group B *Streptococcus* (GBS)-induced lung inflammation, miR-223 was rapidly up-regulated in lung-infiltrating granulocytes at 3-6 h post-GBS infection and this attenuated lung tissue injury 41.

**Figure 2:**
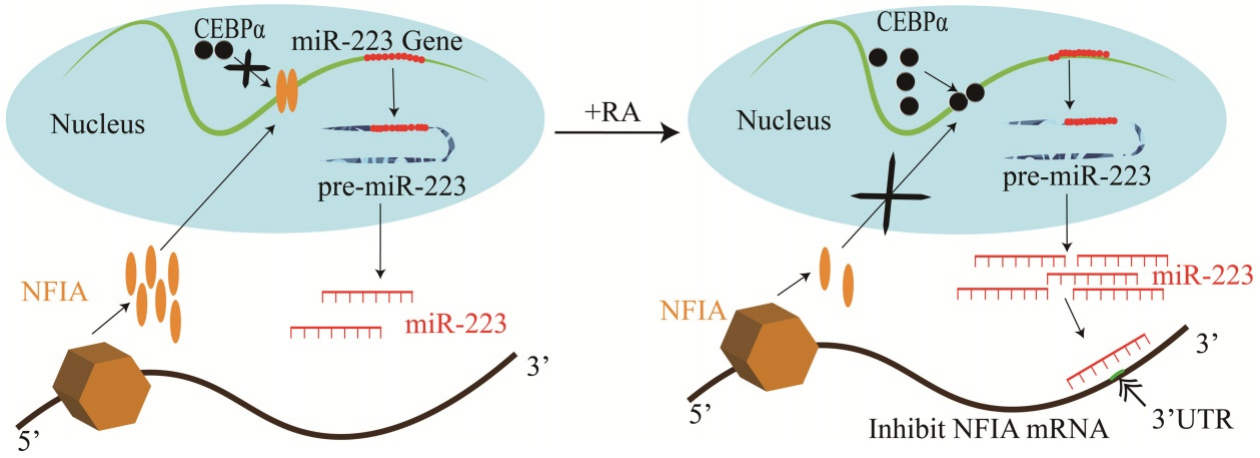
Mechanisms of miR-223 in the regulation of granulocyte differentiation.

Studies of human hematopoietic progenitor cell (HPC) differentiation have revealed that miR-223 is significantly up-regulated by the myeloid transcription factors, PU.1 and C/EBPβ during HPC differentiation into granulocytes and monocytopoiesis 12, 23. However, during erythropoiesis, they are expressed at a low level 23. miR-223 overexpression has been shown to increase granulocytopoiesis, whereas it impairs erythropoiesis and monocyte-macrophage differentiation 23. Other studies have discovered that NFIA could bind to the miR-223 gene promoter and repress its expression during granulocyte differentiation 11, 17. However, retinoic acid triggers C/EBPα to bind to the miR-223 promoter competitively and up-regulates miR-223 expression, inhibiting NFIA expression in a targeted manner and promotes granulocyte differentiation (**Figure 2**) 11, 14.

### Role of miR-223 in macrophage polarization

**Figure 3:**
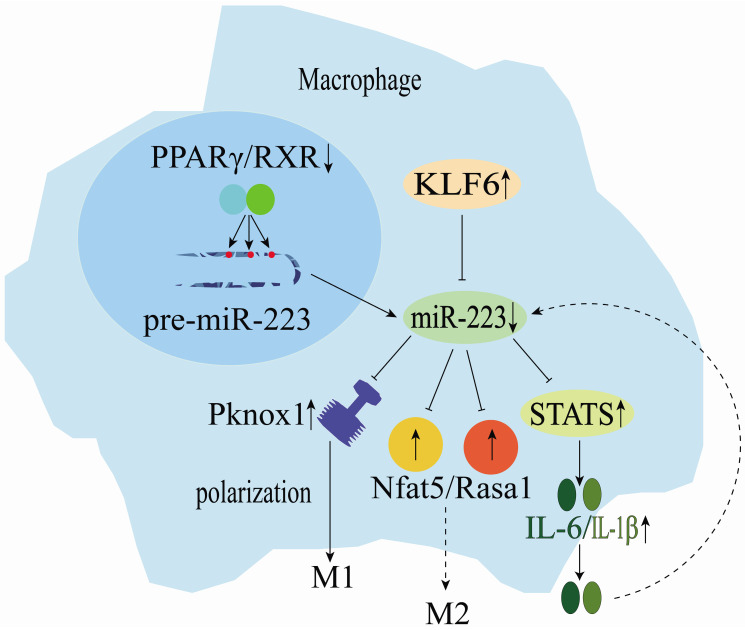
Mechanisms of miR-223 in the regulation of macrophage differentiation.

miRNAs are key regulators of various biological processes and have regulated macrophage (Mø) polarization and promoted inflammatory activities. miR-223 is significantly down-regulated during human monocyte-macrophage differentiation 25. On the other hand, macrophage (M1)-mediated inflammation in adipose and muscle tissues could cause low-grade systemic inflammation development. Macrophages are vital coordinators of immune activity and homeostasis. They could change polarization direction based on temporal and environmental cues and play a central role in promoting host immune defense mechanisms 24. Furthermore, PPARγ regulates the miR-223 expression by directly binding onto PPARγ regulatory elements (PPREs) in the pre-miR-223 promoter (**Figure 3**) 42. Rasa1 and NFAT5 real targets of miR-223 play a crucial role in controlling selective macrophage activation (**Figure 3**) 42. The miR-223 expression could induce the polarization of inflammatory macrophages (M1), as its down-regulation in macrophages reduces the inhibition of STAT genes, promoting the release of LPS-induced interleukin 6 (IL-6) and IL-1β. These cytokines can regulate miR-223 expression negatively and ultimately promote muscle tissue inflammation exacerbation and injury (**Figure 3**) 31, 43, 44. Kruppel-like factor 6 (KLF6) has been identified as a new transcription factor involved in macrophage polarization (**Figure 3**) 16. KLF6 inhibits miR-223 expression by occupying the miR-223 promoter, and KLF6 over-expression has been shown to down-regulate miR-223 expression in macrophages. Furthermore, KLF6-mediated the miR-223 down-regulation in macrophages and has been reported to promote adipose tissue inflammation 15. Moreover, low lncRNA MEG3 expression inhibits M1 macrophage polarization, whereas its deletion could up-regulate miR-223 expression and promote M2 macrophage polarization. High miR-223 expression inhibits TNF receptor-associated factor 6 (TRAF6), suppressing the NF-κB signaling pathway and alleviating myocarditis-associated injury 33. Zhuang et al. 32 showed that miR-223 overexpression could prevent diet-induced adipose tissue inflammation and systemic insulin resistance by inhibiting the Pknox1 gene expression in mice (**Figure 3**). However, macrophages could use microvesicles (MVs) to deliver miR-223, which exerts specific functions in the target cells 45. In summary, miR-223 is a key regulator of the dynamic balance between M1/M2 macrophages and inflammatory diseases.

### Role of miR-223 in dendritic cell differentiation

**Figure 4:**
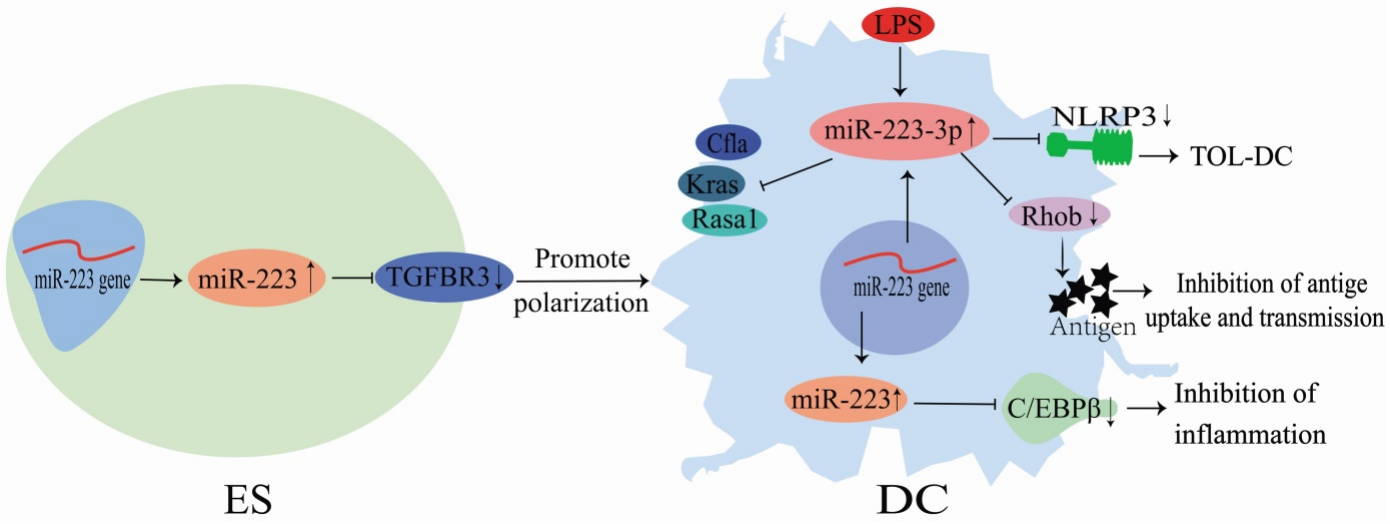
Mechanisms of miR-223 in the regulation of dendritic cell differentiation.

Although miR-223 does not directly act on DCs, it could regulate DC differentiation via several pathways (**Figure 4**). During the differentiation of mouse HSCs into DCs, the miR-223 expression is altered in HSCs, myeloid stem cells and DCs, indicating that miR-223 could play a role in DC differentiation 46. LPS stimulation could up-regulate miR-223-3p expression in DCs, and its high expression could subsequently down-regulate Rasa1, Cfla and Kras mRNA expression and influence immune-related protein regulatory networks 19. Also, miR-223-3p could regulate DC differentiation by binding to Rhob and inhibiting antigen uptake and presentation by DCs 36. Chen et al. 35 showed that miR-223-3p expression was significantly lower in mice with autoimmune myocarditis than in normal mice. Consequently, miR-223-3p could inhibit the NLR family pyrin domain containing 3 (NLRP3) inflammasome to promote the polarization of tolerogenic DCs 35. Furthermore, it could regulate the differentiation and function of mice and human intestinal DCs by targeting C/EBPβ and reducing inflammatory injury (**Figure 4**) 26. Zhu et al. 34 demonstrated that miR-223 directly targeted TGFBR3 to promote the human embryonic stem cells (ESCs) differentiation into DCs. These findings demonstrate that miR-223 is an essential regulator of DC differentiation. It can regulate DC polarization and functions through binding to specific targets, improving tissue inflammation, and preventing the development of inflammatory diseases (**Figure 4**).

### Role of miR-223 in T cell-mediated inflammation

Mature T cells travel through the blood, and reside and proliferate in the T cell zone of peripheral tissues. These cells can be circulated through the body via lymphatics, peripheral blood, and tissue fluids to exert their cellular immune functions. Hosseini et al. 28 showed that miR-223 expression was up-regulated in CD4^+^ T cells during multiple sclerosis. Besides, they could modulate chemokine signaling to promote T helper 17 (Th17) cell expression and suppress regulatory T cell (Treg) differentiation, highlighting a potential miR-223 role in maintaining the Th17/Treg balance. Another study demonstrated that miR-233 is highly expressed in immature CD4^+^ T cells and participates in the proliferation and differentiation of these cells during rheumatoid arthritis 47. Moreover, miR-233 could regulate myeloid DCs (mDCs) to activate and promote the pathological Th17 cells differentiation during autoimmunity 20. Furthermore, miR-223 promotes T helper 1 (Th1) and Th17 cell differentiation and the experimental autoimmune encephalomyelitis (EAE) progression, and its deficiency prevents the infiltration of Th1 and Th17 cells into the spinal cord 27. Additionally, the interaction between miR-223 and SRY-box 11 (SOX11) has gained importance in research towards treating Mantle Cell Lymphoma (MCL) and inflammatory diseases 48. In chicken T cells, both Marek's disease (MD) and overexpressed lnc-GALMD3 result in low miR-223 expression, leading to malignant T cell proliferation 49,50. Taken together, miR-233 regulates T cell proliferation and differentiation and modulates inflammatory diseases by promoting helper T cell proliferation.

### Role of miR-223 in endothelial inflammation

**Figure 5:**
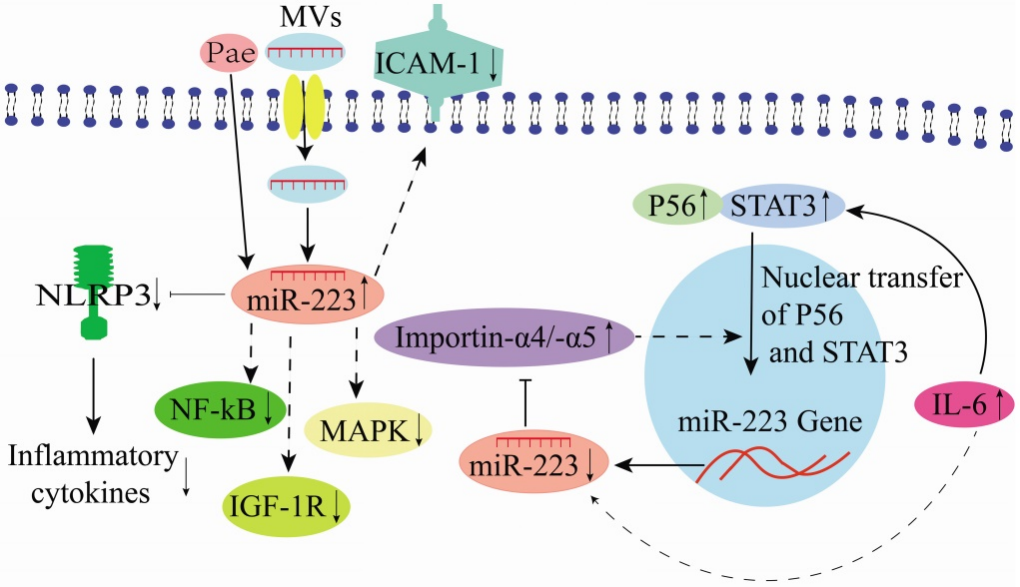
Mechanisms of miR-223 in the regulation of endothelial cells.

Endothelial MVs play a vital role in treating numerous cardiovascular diseases, including atherosclerosis (AS) 21, 51-54. Moreover, in horse, miR-223 in MV participates in immune system regulation by modulating inter leukocyte signaling and inflammatory processes 55. Thrombopoietin could stimulate platelets to release numerous miR-223-expressing MVs 29. Similarly, miR-223 up-regulation has been detected in peripheral MVs (P-MV) in the plasma samples of enteritis, hepatitis, nephritis, and AS patients 29. Platelet-derived miR-223 could be delivered to human umbilical vascular endothelial cells (HUVECs) through P-MVs. Also, it down-regulates the insulin-like growth factor 1 (IGF-1R) expression, promoting HUVEC apoptosis induced by advanced glycation end products (**Figure 5**) 29. Li et al. 56 showed that P-MV-derived miR-223 could inhibit NF-κB and MAPK signaling pathways. Besides, it down-regulates ICAM-1 expression in HUVECs, demonstrating that miR-223 is a critical factor in platelet-derived exosomes that plays essential roles during inflammation and AS (**Figure 5**). Bao et al. 18 demonstrated that IL-6 expression in glomerular endothelial cells (GEnCs) of immunoglobulin A nephropathy (IgAN) patients could induce miR-223 down-regulation. Subsequently, they could promote its binding to importin-α4 and importin-α5, activating GEnCs, and inhibiting the nuclear translocation of P56 and STAT3 (**Figure 5**). STAT3 is critical during the induction of IL-6 in HUVECs, and its expression is positively correlated with IL-6 expression 57, indicating that IL-6/miR-223/importin-α4 (-α5)/STAT3 constitutes a feedback regulatory network in endothelial cells (**Figure 5**).

Intriguingly, the Chinese medicine tree peony bark (Pae) could be a potential therapeutic agent for AS. It could increase monocyte exosome-derived miR-223 57. Additionally, Pae could increase the miR-223 expression level in exosomes derived from the plasma of hyperlipidemic rats to inhibit the NLRP3 inflammasome pathway in endothelial cells, supporting its therapeutic potentials in AS 37, 57. In an endothelial cell study in pigs, miR-223 targeting NLRP3 alleviated inflammation development in porcine endothelial cells and triggered the aorta inflammatory injury 58. Moreover, miR-223 could target β1 integrin to prevent endothelial cell proliferation 59. Differential miR-223 expression in vascular endothelial cell (VEC) MVs influences VEC generation and apoptosis that impacts the functioning of other cells when delivered into the surrounding through VEC-MVs. In contrast, the decrease in miR-223 expression in plasma-derived MVs could attenuate VEC apoptosis 60. Previously, different studies have shown that miR-223-3p is an important regulator of vascular endothelial injury in Kawasaki Disease (KD) 38, 61, 62. It could further reduce vascular endothelial injury by inhibiting IL-6 and TNF-α production 38, and its overexpression could reduce the apoptotic rate of VECs 63. Therefore, there is evidence that some therapeutic agents could modulate miR-223 expression to attenuate endothelial cell injury (**Figure 5**).

### Role of miR-223 in epithelial inflammation

**Figure 6:**
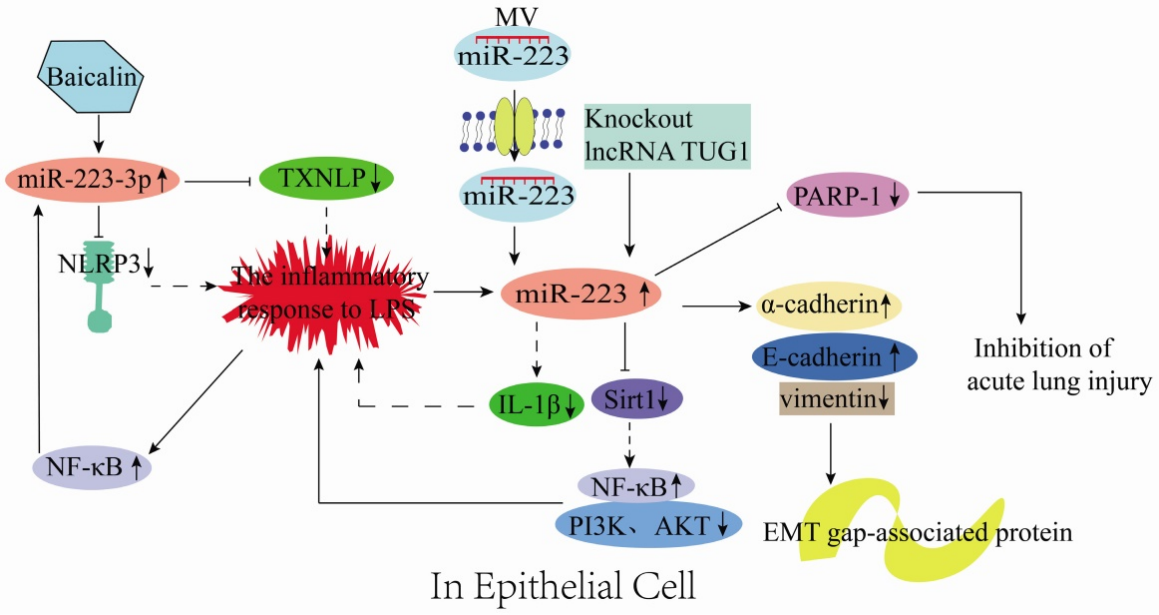
Mechanisms of miR-223 in the regulation of epithelial inflammation.

miR-223 plays essential functions in epithelial cells. For instance, it could control the growth and morphology of mammary epithelial cells 64. Bio-informatic analysis has revealed that NLRP3 is a target of miR-223 and could directly act on renal tubular epithelial cells (RTECs) to induce renal tissue injury in mice 30. Sun et al. 22 reported that in LPS-treated RTECs, baicalin could up-regulate miR-223-3p to inhibit TXNIP and NLRP3 gene expression, resulting in attenuated LPS-induced injury in the proximal tubule epithelial cells (HK-2 cell line) (**Figure 6**). Sirt1 is a direct target of miR-223 in HK2 cells. Notably, low expression of lncRNA TUG1 could increase miR-223 expression, which negatively regulates Sirt1 and reduces PI3K and AKT phosphorylation 39. Also, low expression of lncRNA TUG1 activates the NF-κB signaling pathway and protects HK-2 cells from LPS-induced injury (**Figure 6**) 39. In an *in vitro* model of endometritis, Zhao et al. 65 discovered that LPS treatment could activate the NF-κB signaling that promotes the miR-223 up-regulation in bovine endometrial epithelial cells (BEND). Hence, inhibiting NLRP3 activation and IL-1β production to prevent inflammatory-induced damage (**Figure 6**). Therefore, miR-223 restricts NLRP3 activation and acts as a protective factor during inflammatory responses 65. Liu et al. 40 illustrated that the stromal interaction molecule 1 (STIM1) regulated NLRP3 expression by binding the AACUGA motif in miR-223. On the other hand, silencing STIM1 alleviates influenza A virus (IAV)-induced inflammation in lung epithelial cells by inactivating the NLRP3 and inflammasome by promoting the miR-223 expression 40.

Vesicles are the main structures for information transfer between cells, and 80% of them are derived from epithelial cells. miRNAs are the main components in vesicles and, are transferred between cells along with the vesicles. However, miR-223 in mouse neutrophils could be transferred through MVs to lung epithelial cells and inhibits PARP-1 to prevent acute lung injury (**Figure 6**) 66. In addition, inflammatory mediators could stimulate various signaling pathways to induce the expression of epithelial-mesenchymal transition-related transcription factors (EMT-TFs; e.g., Snail, Zeb, and Twist) and epigenetic regulators (e.g., miRNA, DNA, and histone-modifying enzymes). The expression of miRNA- or DNA-based epigenetic regulators could regulate the expression of genes related to these inflammation signaling pathways, ultimately forming a gene regulatory network 67, 68. Tang et al. 69 reported that miR-223 inhibited cancer cell metastasis by regulating the EMT-related protein expression, which involves up-regulating the epithelial markers (E-cadherin and α-cadherin) and down-regulating the mesenchymal marker vimentin (**Figure 6**). Briefly, the miR-223 expression is up-regulated in epithelial cells, produced by neutrophils, and delivered to epithelial cells via MVs during inflammation. Subsequently, miR-223 regulates the epithelial inflammation process through various signaling pathways to reduce tissue damage (**Figure 6**).

## Role of miR-223 in inflammatory diseases

Recent studies have established that miR-223 could bind to specific target genes to inhibit the production of inflammatory mediators or block inflammation signaling pathways, which protect the body from inflammation-induced injury 11, 70-81. miR-223 plays a vital role in various inflammatory diseases, including acute lung injury, rheumatoid arthritis, enteritis, nervous system inflammation and mastitis (**Table 3**). However, the regulatory networks through which miR-223 modulates immune responses are unclear.

### Role of miR-223 during lung injury

**Figure 7:**
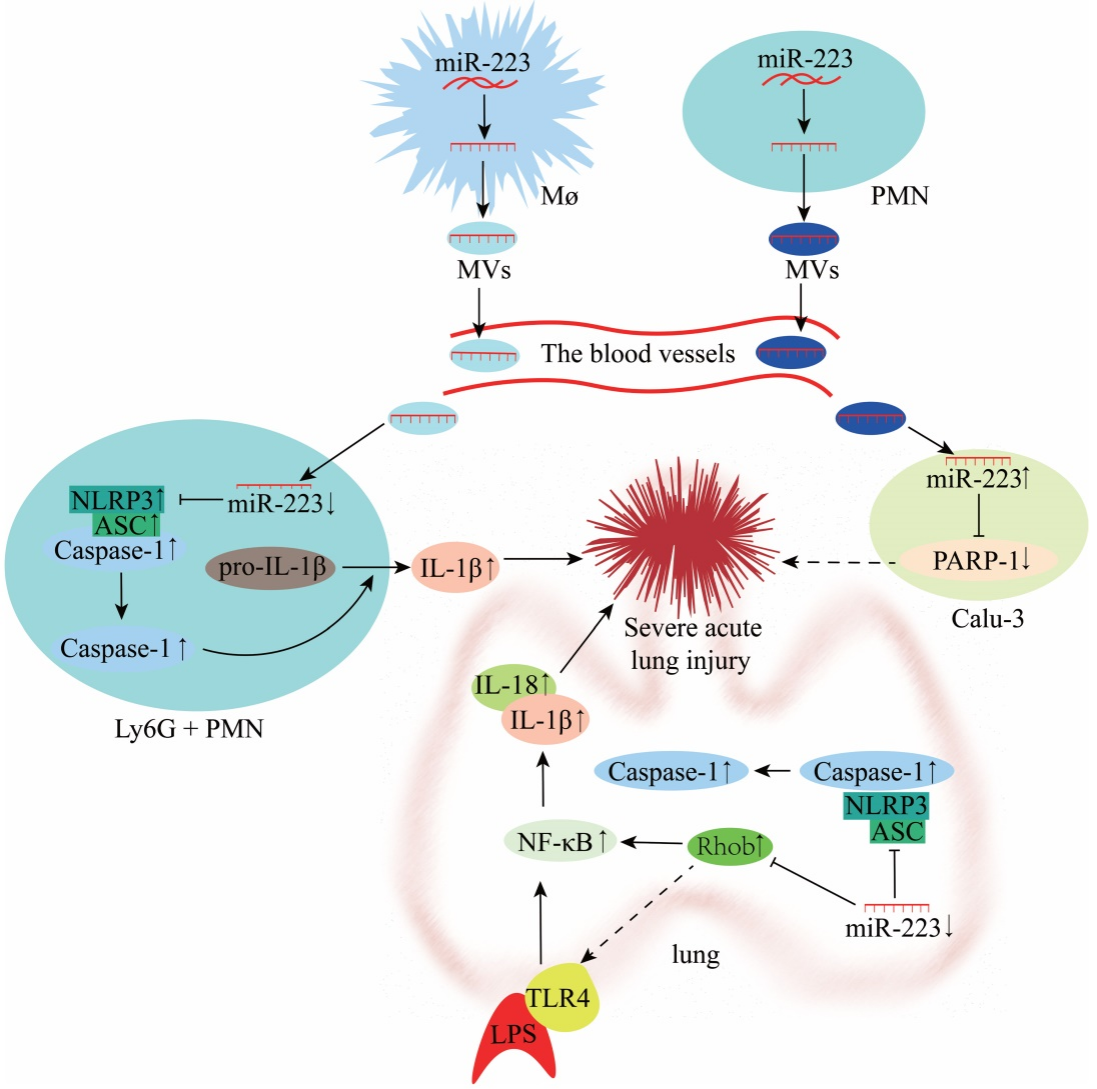
Mechanisms of miR-223 in the modulation of acute lung injury (ALI).

Lung injury involves two processes, namely inflammatory damage and lung fibrosis. Acute lung injury (ALI) is a type of lung injury that has been widely investigated in recent years. Accumulating evidence indicates that miR-223 regulates the NLRP3 inflammasome and plays an important role during ALI 73. However, the TLR4 or NLRP3 inhibitors could impair the anti-inflammatory effect of miR-223 in ALI 11, 72. Moreover, a study showed that the miR-223 response was the fastest in the porcine lung tissue H1N2 infection and highly predicted that NLRP3 could be a miR-223 target, affecting the injury of porcine lung tissue through inflammatory factors 83. In a mouse model of macrophage-mediated lung inflammation, miR-223 regulates macrophage differentiation by targeting the NLRP3 inflammasome. It is transferred by MVs to inhibit lung inflammation (**Figure 7**) 70. Overexpression of miR-223 can directly inhibit NF-κB in bronchial epithelial cells to alleviate lung inflammation 84. Furthermore, miR-223 expression was reported to be down-regulated in LPS-treated A549 cells (lung adenocarcinoma cells) 72. *In vitro* experiments also demonstrated that decreased miR-223 expression resulted in diminished inhibition of RHOB, NLRP3 inflammasome and TLR4/NF-κB pathway, exacerbating lung injury (**Figure 7**) 72, 85. In a mouse study of hippocampal inflammation, aerobic exercise can induce miR-223 expression in the hippocampus, which negatively regulates the TLR4/MyD88-NF-κB pathway to improve inflammation-induced injury 86. miR-223 can also alleviate neutrophilic airway inflammation by inhibiting NLRP3 inflammasome and IL-1β release 87. However, miR-223 was reported to attenuate ALI induced by mitochondrial damage-associated molecular patterns (MTDs) by limiting the differentiation of bone marrow-derived Ly6G^+^ neutrophils and inhibiting NLRP3 inflammasome activity and IL-1β production 71. On the other hand, miR-223 deficiency results in persistent NLRP3 and IL-1β activation and exacerbates lung injury 88. MV-mediated transfer of miR-223 from neutrophils to lung epithelial cells (Calu-3) attenuates ALI via inhibition of poly (ADP-ribose) polymerase-1 (PARP-1) (**Figure 7**) 89, 90. However, the miR-223 expression is significantly down-regulated during lung fibrosis, and this is attributed to the antioxidative properties of the amino acid hydroxyproline (HYP) 91. miR-223 can alleviate ALI by regulating the process of inflammation, which serves as a new potential therapeutic target and prognostic marker for ALI (**Figure 7**).

### Role of miR-223 in rheumatoid arthritis

Rheumatoid arthritis (RA) is a chronic inflammatory disease. Studies have demonstrated that miR-223 is significantly higher in the plasma, serum and peripheral mononuclear cells (PBMCs) of RA patients than those of controls, indicating that miR-223 could be associated with the development, progression and severity of RA 92, 93. The reliability of the biological functions of miR-223 in RA has been confirmed by accurately measuring the AUC value, and sensitivity and specificity of miR-223 94. Also, other studies have shown that the expression of miR-223 in PBMCs and plasma is positively correlated with the level of rheumatoid factor (RF) in RA patients 95-98. miR-223 is differentially expressed between the PBMCs and plasma in RA patients, suggesting that miR-223 could be a biological marker for RA diagnosis 95-98.

**Figure 8:**
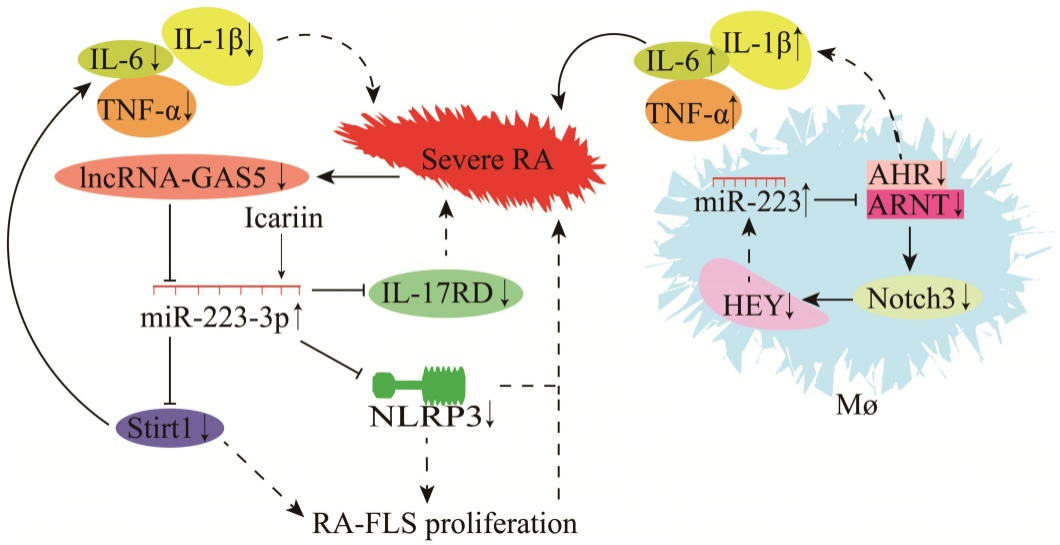
Mechanisms of miR-223 in the modulation of rheumatoid arthritis (RA).

The role of miR-223 in RA is highly complex (**Figure 8**). miR-223 is significantly up-regulated during RA in mice. Some target genes regulated by miR-223 include actin alpha 1 (ACTA1), (ACVR2A), cholecystokinin B receptor (CCKBR), dual-specificity phosphatase 10 (DUSP10), forkhead box O1 (FOXO1), heat shock protein 90 beta family member 1 (HSP90B1), interleukin 6 cytokine family signal transducer (IL6ST), inositol polyphosphate-5-phosphatase B (INPP5B), MX dynamin-like GTPase 1 (MX1), protein tyrosine phosphatase non-receptor type 2 (PTPN2), and tyrosine 3-monooxygenase/tryptophan 5-monooxygenase activation protein gamma (YWHAG) 99. According to a recent study in the arthritis fibroblast cell line MH7A, IL-17 receptor D (IL-17RD) was identified as a target gene of miR-223-3p 100. Overexpression of miR-223-3p could down-regulate IL-17RD expression and alleviate RA-induced injury 100, 101. Furthermore, the mouse model also showed that icariin could inhibit NLRP3 by up-regulating miR-223-3p expression, reducing the apoptosis of RA joint fibroblast-like synovial cells (RA-FLSCs), and attenuates RA-induced injury 102, 103. Sirt1 is also a target gene of miR-223-3p, and lncRNA-GAS5 is a molecular sponge for miR-223-3p 82, 104. Down-regulation of lncRNA-GAS5 during RA leads to the up-regulation of miR-223-3p and down-regulation of Sirt1. It decreases the secretion of TNF-α, IL-6, IL-8, and IL-1β and reduces RA-FLSC apoptosis, hence slowing RA progression 82, 104. Macrophages are critical players in RA pathogenesis, and these are the most abundant inflammatory cells in RA. Ogando et al. 74 discovered that miR-223 was significantly up-regulated in the macrophages of RA patients. Increased miR-223 expression inhibited ARNT protein synthesis (a co-receptor for AHR) 105 and prevented AHR/ARNT-mediated pro-inflammatory cytokine expression. AHR agonists could suppress the gene expression of pro-inflammatory cytokines (TNF-α, IL-1β, and IL-6), Notch3, and HEY. A low level of HEY could up-regulate miR-223 expression in macrophages (**Figure 8**) 74. These findings demonstrate that miR-223 regulates RA progression by increasing the macrophage sensitivity to pro-inflammatory cytokines and dampening their response to anti-inflammatory signals during RA 74.

### Role of miR-223 in enteritis

Enteritis could be classified into bacterial enteritis, parasitic enteritis and viral enteritis based on pathogens. Bacteria cause it, and microbes and viruses in the intestine could also induce enteritis 106, 107. Several studies have demonstrated that miR-223 is highly expressed in the intestinal tissues, serum, and feces of ulcerative colitis (UC) and inflammatory bowel disease (IBD) patients, suggesting that miR-223 is involved in its regulation and could serve as a biomarker for UC and IBD 108-110. Besides, NLRP3 inflammasome expression in mice and humans is gradually elevated as IBD exacerbates 111, 112, demonstrating that the expression of inflammasomes (NLRP1, NLRP2 and NLRP3) dictates the severity of IBD 113.

**Figure 9:**
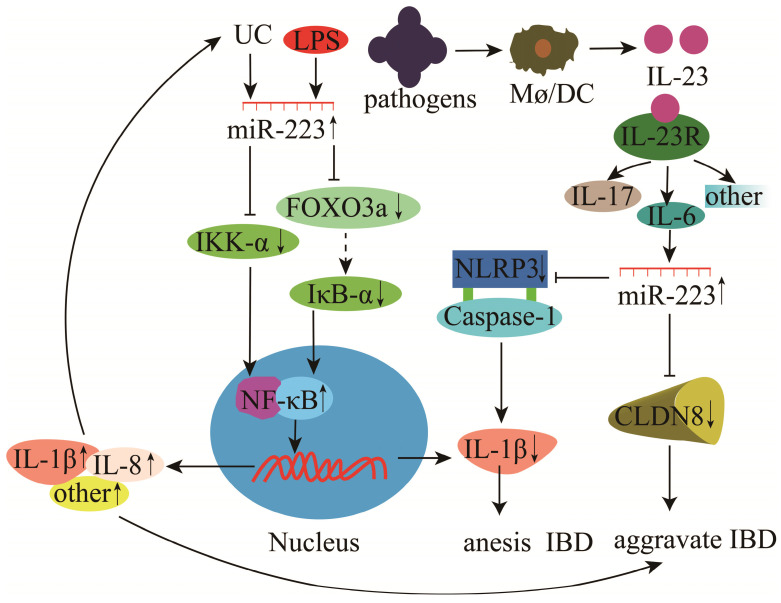
Mechanisms of miR-223 in the modulation of enteritis.

miR-223 plays a vital role in the development and progression of IBD and UC. Studies have found that miR-223 expression is significantly increased in the mucosal biopsy tissues of UC patients 114. miR-223 regulates UC by inhibiting the expression of IKK-α, a negative regulator of NF-κB, and promotes the release of p56 and pro-inflammatory cytokines (IL-1β and IL-8) (**Figure 9**) 75. In the case of IBD, LPS stimulation induces the inhibition of FOXO3a by miR-223, which subsequently down-regulates IκB-α and activates the NF-κB signaling pathway to release pro-inflammatory cytokines, thus promoting IBD progression (**Figure 9**) 115. Neudecker et al. 76 showed that miR-223 expression was up-regulated during experimental IBD in mice. The increase in miR-223 expression caused the down-regulation of NLRP3 inflammasome and IL-1β secretion, reducing IBD severity (**Figure 9**) 76. In the dextran sodium sulfate (DSS) colitis model, miR-223 can alleviate intestinal inflammation by reducing the release of inflammatory mediators via inhibition of IL-6 and STAT3 signaling 116. Claudin-8 (CLDN8) is a member of the claudin multigene family and the primary tight junction protein. CLDN8 expression has been significantly down-regulated in the inflamed colonic mucosa of IBD patients and mice with trinitrobenzene sulfonic acid (TNBS)-induced colitis. Its level could be restored in colitic mice treated with IL-23 antibody. However, miR-223 could bind to CLDN8, and its up-regulation exacerbates IBD (**Figure 9**). Therefore, the interaction among IL-23, miR-223, and CLDN8 could serve as a new therapeutic strategy for IBD 77.

### Role of miR-223 in central nervous system inflammation

**Figure 10:**
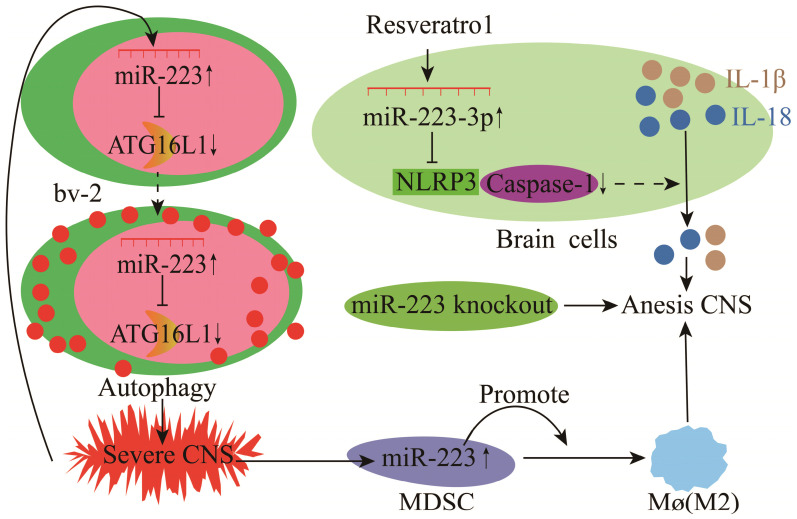
Mechanisms of miR-223 in the modulation of experimental autoimmune encephalomyelitis (EAE).

Inflammation of the central nervous system (CNS) mainly includes EAE, multiple sclerosis (MS), and bacterial meningitis 117-119. Microglial cells (bv-2) are resident macrophages of the CNS and have critical physiological functions in attenuating CNS inflammation and maintaining tissue homeostasis 120, 121. miR-223 deficiency increases the autophagy of resting microglia and microglia in the brain, which significantly reduces demyelination and improves CNS inflammation and the clinical symptoms of EAE 78. Notably, normal cell proliferation and autophagy are metabolic processes required to maintain homeostasis, and abnormal proliferation and autophagy could cause inflammatory diseases. Different, studies have shown that miR-223 could inhibit P21 by inhibiting NFIA expression, resulting in abnormal microglia proliferation 78, 122. In mouse EAE and MS, the miR-223 expression was significantly increased as the disease duration increased 123, 124. Deleting miR-223 (miR-223^-/-^) could significantly delay EAE onset, alleviate spinal injury and decrease neurological symptoms in mice, demonstrating that miR-223 is a potential marker and therapeutic target for EAE (**Figure 10**) 123, 124. Also, miR-223 promotes MS progression by inhibiting bv-2 autophagy via the down-regulation of ATG16L1 (**Figure 10**) 78. Galloway et al. 125 found that miR-223 effectively regulated M2 polarization and promoted bone marrow activation and CNS remyelination. These findings demonstrate a critical pathophysiological relationship between miR-223 and MS, and other neurodegenerative diseases, thus providing new insights into MS diagnosis and treatment 78. During BM, resveratrol could modulate the miR-223-3p/NLRP3 pathway to inhibit downstream caspase-1 activation and IL-1β and IL-18 processing in neurons and bv-2 cells, protecting cortical neurons from inflammatory damage and death (**Figure 10**) 79. However, findings from bv-2 cells have shown that lncRNA GAS5 and NLRP3 are ceRNAs that sponge miR-223-3p and a high expression of lncRNA GAS5 promotes NLRP3 activation and pro-inflammatory cytokine release 126.

Myeloid-derived suppressor cells (MDSCs) play essential regulatory and effector functions in MS and EAE. miR-223 knockout (miR-223^-/-^) mice have increased MDSCs in the spleen and spinal cord and milder EAE, supporting the critical role of miR-223 in the regulation of MDSCs during EAE and MS and further highlights the possibility of miR-223 as a new therapeutic agent 127.

### Role of miR-223 in mastitis

Mastitis could be caused by several factors, including pathogen infection and environmental changes and has complex pathogenesis. *Staphylococcus aureus*,* Streptococcus. uberis*,* and Escherichia. coli* are common causative agents of mastitis 128. LPS is a surface component of E. coli that induces inflammation. Hao et al. 129 demonstrated that miR-223 was significantly up-regulated during LPS-induced mammary epithelial inflammation in mice. In addition, miR-223 expression was 2.5-3 times higher in the mammary tissue of cows with mastitis than that of healthy cows 130-134, suggesting that it could be a biomarker for mastitis 135, 136.

**Figure 11:**
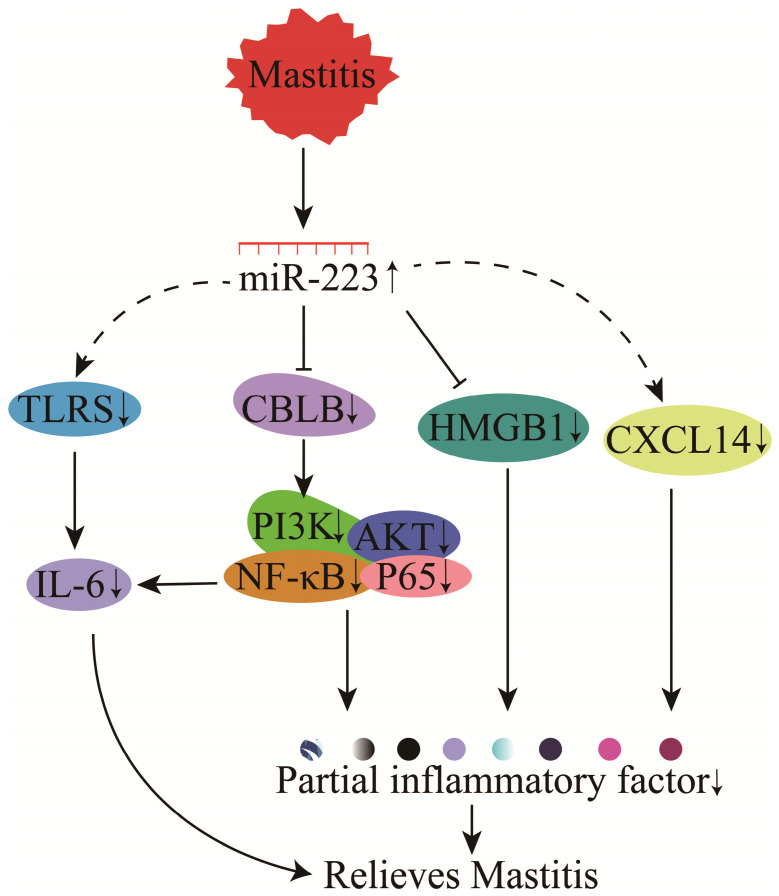
Mechanisms of miR-223 in the modulation of mastitis.

The molecular mechanism of miR-223 in regulating mastitis has been investigated in cows, humans, and mice. Moreover, miR-223 in MVs released by platelets could effectively inhibit the carcinogenesis of human breast cells 137. miR-223 could participate in TLR signaling and down-regulate IL-6 protein expression (not gene expression) to inhibit inflammation (**Figure 11**) 138. Studies of *Staphylococcus. aureus* or lipoteichoic acid (LTA)-induced mastitis have identified CBLB as a target gene for miR-223 81, 139. Bovine miR-223 could target CBLB and inhibit the PI3K/AKT/NF-κB signaling pathway to suppress IL-6 expression and attenuate inflammation (**Figure 11**) 81. Furthermore, miR-223 could inhibit CXCL14 and regulate bovine mastitis through a series of complex gene regulatory networks (GRNs) (**Figure 11**) 140.

High mobility group box 1 (HMGB1) is involved in the pathogenesis of multiple inflammatory diseases, including bovine mastitis 141. The functional SNP in the 3'-UTR of HMGB1 could affect its binding to miR-223, thereby influencing bovine mastitis regulation through miR-223 (**Figure 11**) 80. Also, miR-223 has demonstrated protein-protein interactions with the predicted candidate genes (F-box protein 30 (FBXO30), SMAD specific E3 ubiquitin-protein ligase 2 (SMURF2), F-box and WD repeat domain containing 7 (FBXW7), and ubiquitin-like modifier activating enzyme 2 (UBA2), suggesting that miR-223 could regulate mastitis through bacterial invasion, endocytosis, antigen processing, immune response, and TGF-β and MAPK signaling in mammary epithelial cells 142-144.

## Conclusions and future perspectives

miR-223 is a multifunctional miRNA regulated by several transcription factors, and its expression is significantly elevated during cellular or tissue inflammation. miR-223 regulates immune cell proliferation, differentiation and polarization, and has immunomodulatory effects in certain tissues. It also serves as a messenger and a regulator of inflammation in immune and resident cells. Furthermore, it plays a critical role in cell-cell, cell-tissue, and cell-tissue-inflammatory disease interactions. Elucidation of its molecular mechanisms in immune modulation could provide insights in developing early diagnosis or treatment for inflammatory diseases. Although several miR-223 target genes have been identified in humans and animals have effectively mediated the regulatory functions of miR-223, some of these functional studies have only been conducted using *in vitro* setups or at the cellular level. Thus, it is imperative to establish* in vivo* studies to validate these findings. In addition, the miR-223 target genes could be used to construct a detailed multi-species miR-223 regulatory network based on their sequence homology to identify precise miR-223-targeting drugs. These drugs could range from bioactive ingredients to DNA, mRNA, and translated proteins, and drugs identified using this method have high precision. Lastly, the drugs could exhibit better efficacy in treating inflammatory diseases, especially autoimmune disorders.

## Figures and Tables

**Table 1:** Change in miR-223 expression during the inflammation of various cell types

Cell type	miR-223 expression	Affected tissue	Reference
Granulocytes	Significantly up-regulated	Lung tissue	13, 23
Macrophages	Significantly down-regulated	Fat and muscle tissues	15, 24, 25
Dendritic cells	Down-regulated	Small intestinal tissue	19, 26
T cells	Highly expressed	Nerve tissues	20,27,28
Endothelial cells	Mass delivery via microvesicles highly expressed	Tissues supplied by arteries	21, 29
Epithelial cells	Up-regulated	Kidney tissues	22, 30

**Table 2:** Intracellular targets and functions of miR-223

Cell	Target	Function	Reference
Granulocytes	NFIA, C/EBPα, and Mef2c	Regulates granulocyte proliferation, and differentiation	11, 13, 14
Macrophages	STATS, Pknox1, and TRAF6	Regulates macrophage differentiation, polarization, and pro-inflammatory cytokine release, and promotes NF-kB-induced inflammatory injury	31-33
Dendritic cells	NLRP3, C/EBPβ, TGFBR3, MR, Rhob, Rasa1, Cfla, and Kras	Regulates dendritic cell functions and influences immune-related protein networks	19, 26, 34-36
Endothelial cells	importin-α4, importin-α5, NLRP3, and IL-6	Attenuates endothelial cell injury	18, 37, 38
Epithelial cells	NLRP3, Sirt1, and ST1M1	Attenuates epithelial cell injury	30, 39, 40

**Table 3:** Target and role of miR-223 in various inflammatory diseases

Inflammatory disease	Target	Function	Reference
Acute lung injury (ALI)	RHOB, NLRP3, and PARP-1	Alleviates ALI	11,70-73
Rheumatoid arthritis (RA)	ARNT and Stirt1	Alleviates RA	74,82
Enteritis	NLRP3, CLDN8, and IKK-α	Alleviates enteritis	75, 76
Nervous system inflammation	ATG16L1 and NLRP3	Alleviates nervous system inflammation	78, 79
Mastitis	HMGB1 and CBLB	Alleviates mastitis	80, 81

## Abbreviations

Sirt1: sirtuin1
DCs: dendritic cells
RISC: RNA-induced silencing complex
qPCR: quantitative polymerase chain reaction
NFIA: nuclear factor I A
Mef2c: myocyte enhancer factor 2C
LPS: lipopolysaccharide
GBS: Group B Streptococcus
HPC: hematopoietic progenitor cell
Mø: macrophage
PPREs: PPARγ regulatory elements
MVs: microvesicles
IL-6: interleukin 6
KLF6: Kruppel-like factor 6
TRAF6: TNF receptor associated factor 6
NLRP3: NLR family pyrin domain containing 3
SOX11: SRY-box 11
MCL: Mantle Cell Lymphoma
MD: Marek's disease
ESCs: embryonic stem cells
Th17: T helper 17
Treg: regulatory T cell
EAE: experimental autoimmune encephalomyelitis
MCL: Mantle Cell Lymphoma
AS: atherosclerosis
P-MV: peripheral microvesicles
IGF-1R: insulin-like growth factor 1
HUVECs: human umbilical vascular endothelial cells
GEnCs: glomerular endothelial cells
IgAN: immunoglobulin A nephropathy
VEC: vascular endothelial cell
KD: Kawasaki Disease
RTECs: renal tubular epithelial cells
BEND: bovine endometrial epithelial cells
STIM1: stromal interaction molecule 1
IAV: influenza A virus
EMT-TFs: epithelial-mesenchymal transition-related transcription factors
ALI: Acute lung injury
MTDs: mitochondrial damage-associated molecular patterns
RA: Rheumatoid arthritis
PBMCs: peripheral mononuclear cells
RF: rheumatoid factor
ACTA1: actin alpha 1
ACVR2A: A receptor type 2A
CCKBR: cholecystokinin B receptor
DUSP10: dual specificity phosphatase 10
FOXO1: forkhead box O1
HSP90B1: heat shock protein 90 beta family member 1
IL6ST: interleukin 6 cytokine family signal transducer
INPP5B: inositol polyphosphate-5-phosphatase B
MX1: MX dynamin like GTPase 1
PTPN2: protein tyrosine phosphatase non-receptor type 2
YWHAG: tyrosine 3-monooxygenase/tryptophan 5-monooxygenase activation protein gamma
RA-FLSCs: RA joint fibroblast-like synovial cells
DSS: dextran sodium sulfate
CLDN8: Claudin-8
TNBS: trinitrobenzene sulfonic acid
IBD: inflammatory bowel disease
UC: ulcerative colitis
CNS: Inflammation of the central nervous system
GRNs: gene regulatory networks
FBXO30: F-box protein 30
SMURF2: SMAD specific E3 ubiquitin protein ligase 2
FBXW7: F-box and WD repeat domain containing 7
UBA2: ubiquitin like modifier activating enzyme 2
HMGB1: High mobility group box 1
